# Rationale and Application of PEGylated Lipid-Based System for Advanced Target Delivery of siRNA

**DOI:** 10.3389/fphar.2020.598175

**Published:** 2021-01-20

**Authors:** Xuemei Ge, Lijuan Chen, Bo Zhao, Weien Yuan

**Affiliations:** ^1^ Department of Food Science and Technology, College of Light Industry Science and Engineering, Nanjing Forestry University, Nanjing, China; ^2^ Engineering Research Center of Cell and Therapeutic Antibody, Ministry of Education, and School of Pharmacy, Shanghai Jiao Tong University, Shanghai, China

**Keywords:** siRNA, PEGylated lipid-based nanoparticles, target, delivery systems, mechanism

## Abstract

RNA interference (RNAi) technology has become a powerful tool in application of unraveling the mechanism of disease and may hold the potential to be developed for clinical uses. Small interfering RNA (siRNA) can bind to target mRNA with high specificity and efficacy and thus inhibit the expression of related protein for the purpose of treatment of diseases. The major challenge for RNAi application is how to improve its stability and bioactivity and therefore deliver therapeutic agents to the target sites with high efficiency and accuracy. PEGylated lipid-based delivery system has been widely used for development of various medicines due to its long circulating half-life time, low toxicity, biocompatibility, and easiness to be scaled up. The PEGylated lipid-based delivery system may also provide platform for targeting delivery of nucleic acids, and some of the research works have moved to the phases for clinical trials. In this review, we introduced the mechanism, major challenges, and strategies to overcome technical barriers of PEGylated lipid-based delivery systems for advanced target delivery of siRNA *in vivo*. We also summarized recent advance of PEGylated lipid-based siRNA delivery systems and included some successful research works in this field.

## Introduction

Since antisense oligonucleotides (ASOs) have proven to be efficient drugs for treatment of certain diseases by complementary base pairing to target mRNA to inhibit protein expression or splicing pre-mRNA to mature mRNA (Stephenson and Zamecnik, 1978; Altman, 2012), till now, some of the oligonucleotide drugs were approved for clinical uses, such as Vitravene (fomivirsen) for the treatment of cytomegalovirus retinitis and Kynamro (mipomersen) for the treatment of familial hypercholesterolemia, and the results are encouraging (Swayze, 2010; Castanotto, 2017; Geary, 2017). Small interfering RNA (siRNA), which is consisted of 21–23 nucleotides, has emerged as a powerful tool either in fundamental research or potential medicines for clinical uses in recent decades (Wittrup and Lieberman, 2015). These oligonucleotides may target mRNA with high specificity and induce degradation of the targeted mRNA to regulate the expression of related protein by RNA interfering (Reynolds et al., 2004; Bruno, 2012; Wittrup and Lieberman, 2015). However, these oligonucleotides are unstable and easy to be cleared when exposed to body fluid after administration, and also, the efficacy and accuracy of delivering siRNA to target site is another challenge for its application. How to overcome these drawbacks and barriers is still challenging (Ho et al., 2016). Lipid-based delivery system plays an important role in drug developing and may encapsulate siRNA and offer a feasibility to help siRNA to reach the desired targets. ONPATTRO™ (patisiran) has been approved by the United States Food and Drug Administration (FDA) as the first RNAi-based drug to be used clinically in 2018. More siRNA candidates are now in clinical trials (Hu et al., 2019; Jayesh et al., 2019; Leung et al., 2019).

Due to the nature of these bio-molecules and obstacles existing in the delivery pathways, efficient delivery system is needed to help these therapeutic agents to reach the target sites safely and accurately (Raye et al., 2018). Many strategies were developed for siRNAs delivery such as viral vectors, physical methods (hydrodynamic injection, particle bombardment, and electroporation), chemical methods, and polymer- or lipid-based delivery systems (Liu et al., 1999; Davis, 2007; Patil and Panyam, 2009). Lipid-based delivery system has proven to be effective in delivering various kinds of drugs such as chemical drugs, proteins, and oligonucleotides. It holds the advantages of good biocompatibility, low toxicity, and easiness to be modified by chemical reaction to immobilize functional components. These nanoparticles are easy to be scaled up and used. However, liposomes without further modification were easy to be captured nonspecifically such as the reticuloendothelial system (RES) after administration. Some lipid-based delivery systems for siRNA involve in usage of cationic lipids, which may help with cell up-taking and endosome escaping efficiency; these nanoparticles can be attached to negatively charged cell membrane surface and thus induce nonspecific absorption (Bouxsein et al., 2007; Fitzgerald et al., 2019; Huang and Fish, 2019). In recent years, a variety of lipid-based nanoparticles were developed to enhance the delivery efficiency of siRNA (Thi et al., 2014; Li et al., 2020). To address this issue, PEGylation was widely used to increase the circulation time and efficiency *in vivo*. In previous work, the PEGylation on the surface of liposome for siRNA was well introduced to illustrate the effects in different kinds of liposomes (Yuen et al., 2013; Xia et al., 2015). It has proven that conjugation of PEG on the surface of liposomes could enhance the half-life time due to their sterical stabilization, which could contribute to the van der Waals interactions between the protein/lipid bilayer surface or steric repulsion of PEG polymer layer. It could avoid nonselective adhesion, maximization of selective uptake by conjugation of specific ligand. In this work, we summarize the mechanism and delivery pathway of siRNA, barriers of siRNA target delivery, rationale design of lipid-based nanoparticles, and challenge and solution in siRNA delivery, which were associated with PEGylation.

## Mechanism of siRNA

**FIGURE 1:**
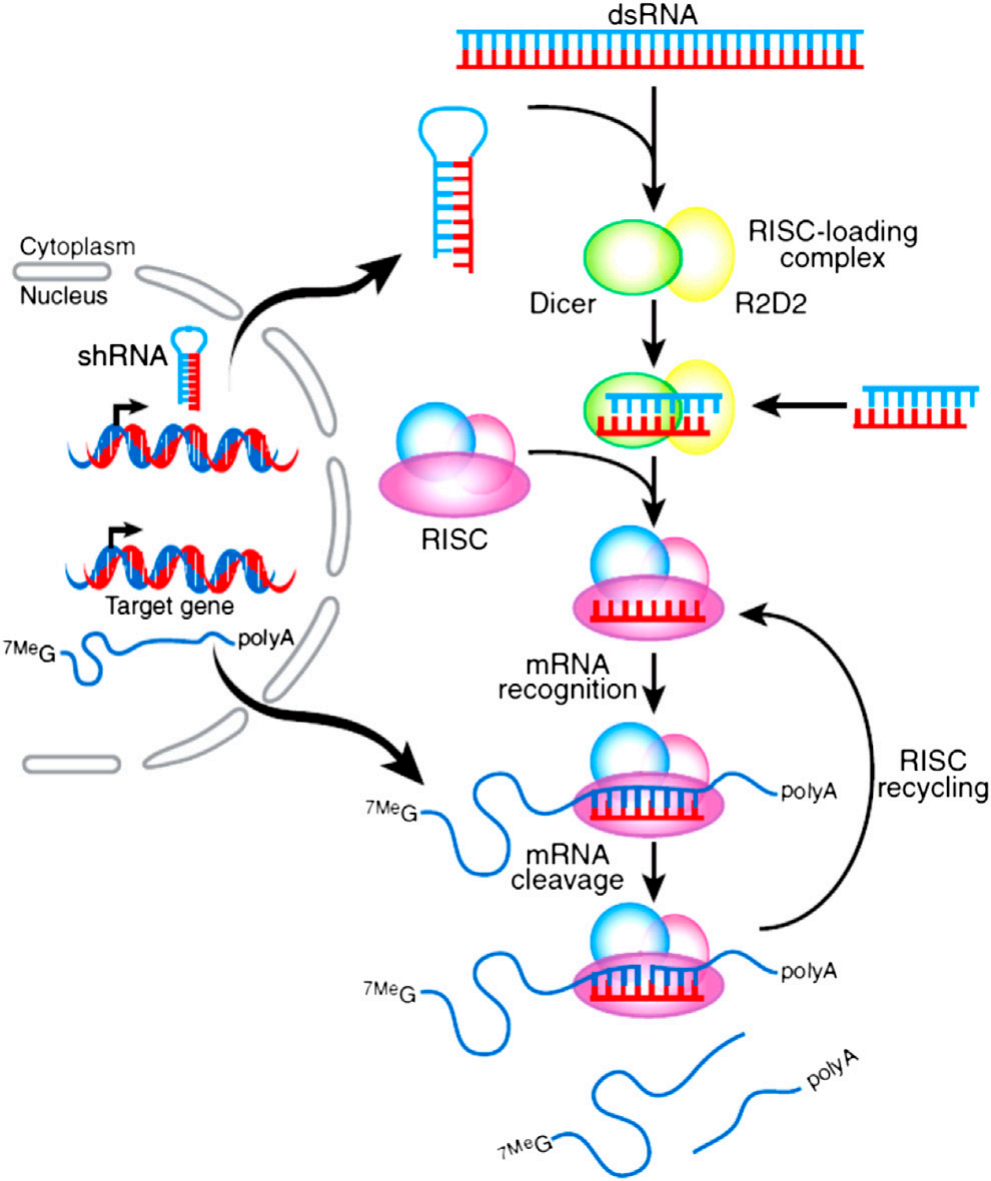
Mechanism of siRNA in silencing target gene. Precursor RNA was combined with Dice R2D2 binding protein to produce siRNA and introduced into RISC. The active RISC containing antisense strand RNA cleavages the target mRNA to induce the silencing effect with several rounds of this action (Dominska and Dykxhoorn, 2010).

Mechanisms of RNA interference pathway begin with the processing of double-stranded RNA (dsRNA) into short RNA duplexes as shown in Figure 1. siRNA was derived from longer precursor RNA, which may be processed in the cytoplasm by one of the RNase III named Dicer with R2D2 dsRNA binding protein. With this process, the siRNA molecules are introduced into the RNA-induced silencing complex (RISC), the endonuclease Argonaute-2 cuts the sense strand to produce reactive RISC which contains antisense strand RNA. RISC complementarily binds to target mRNA to induce the cleavage and inhibit the expression of related protein. The formed siRNA-loaded RISC could be recycled for several rounds in mRNA recognition, mRNA cleavage, as shown in Figure 1 (Reynolds et al., 2004; Miyagishi and Taira, 2005; Wolfrum et al., 2007). siRNA is not easy to be delivered into target cells after administration due to poor stability both *in vitro* and *in vivo*, larger molecular size, and negative charges; these factors limit siRNA to be used for clinical treatments. The development of efficient delivery system is the keypoint for the successful application of siRNA (Akashi et al., 2005; Abreu-Goodger et al., 2008; Dominska and Dykxhoorn, 2010).

## Delivery Pathways of siRNA Based on Lipid

siRNA is unstable and easy to be cleared during the *in vivo* circulation. It is reported that naked siRNA is subject to degradation and the half-life is no more than 5 min in plasma (Layzer et al., 2004). It has demonstrated that modification of siRNA with lipid could improve plasma bioavailability. The lipophilic molecules were conjugated to chemically modified siRNA targeting apolipoprotein B (apoB). The lipophile-conjugated siRNA could bind to lipoprotein particles efficiently to promote intracellular uptake. These conjugates are stable in both human and mouse serum for over 48 h. The fatty-acid conjugates with a longer, saturated, alkyl chain could significantly lower apoB mRNA level(Wolfrum et al., 2007). The gene silencing activity of lipophilic siRNAs was reported to also affect by the length of the linker between siRNA and lipophilic group (Petrova et al., 2012). The conjugation of lipid to siRNA could provide a promising way for its therapeutic applications (Kubo et al., 2013). Lipid-based system attracts much attention to improve the stability of siRNA since the discovery of RNAi technology and quite a lot of commercial lipid transfect reagents were developed for research work (Felgner, 1991; Hamby, 1995; Dalby et al., 2004). To achieve the efficient delivery of these therapeutic siRNAs to the targeted cells, the delivery pathways of the nanoparticles should be taken into consideration. Although some siRNAs are injected locally, such as age-related macular degeneration (AMD) treatment, most of these therapeutic agents need to be administrated systematically to circulate and reach the target cells (Fattal and Bochot, 2006; Nguyen et al., 2008; Whitehead et al., 2009; Yang et al., 2013). PEGylation of lipid-based nanoparticles is effective to help these vectors for high efficacy.

**FIGURE 2:**
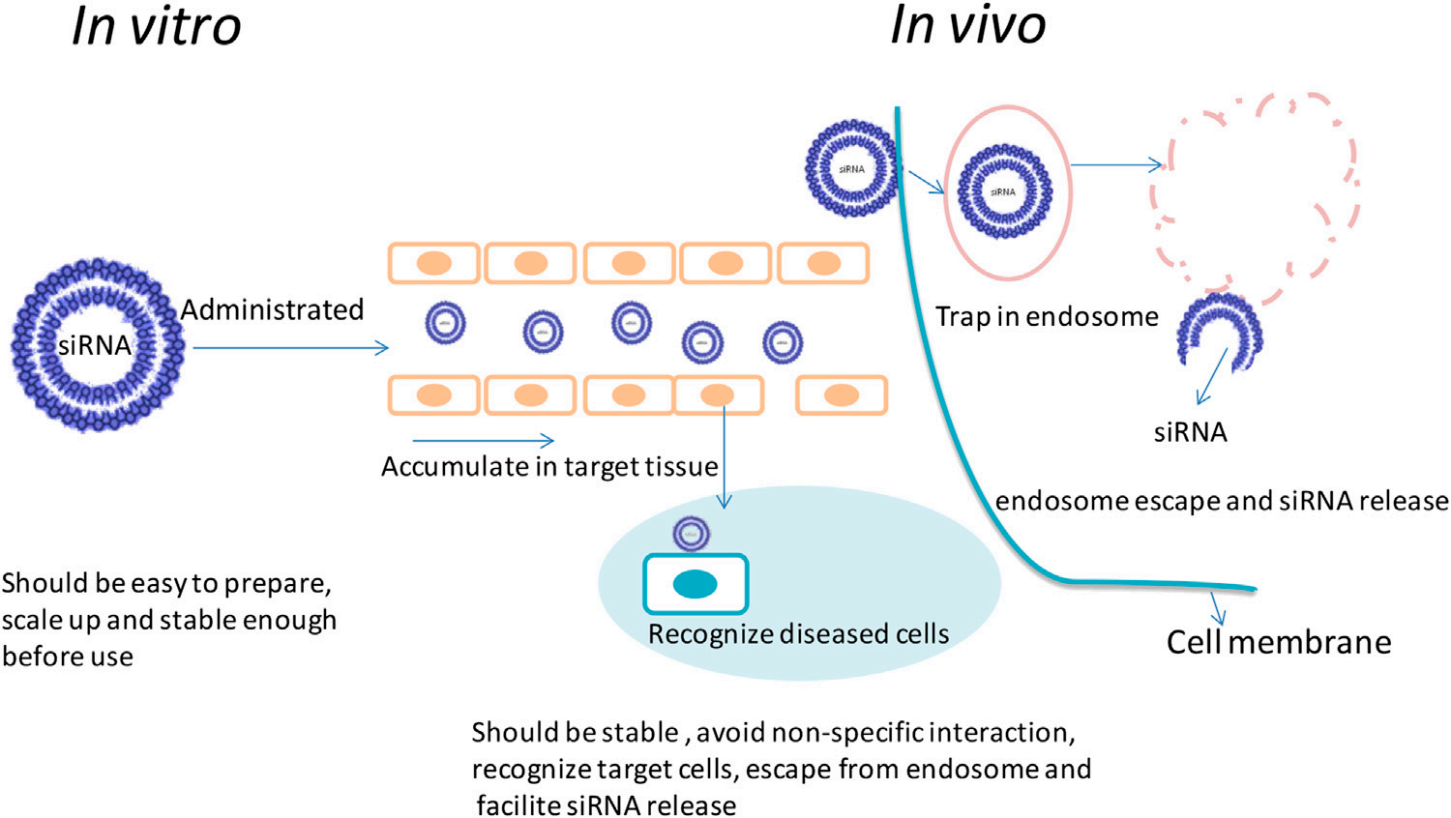
Main biological delivery pathway of siRNA. siRNAs can be encapsulated into nanosize particles with desired sizes to avoid renal clearance as well as reach the target tissues. It should be easy to prepare. The nanoparticle should be strong enough to avoid association and protect siRNA from degradation; before reaching the cytoplasm, it should also offer the mechanism to escape from endosome and facilitate sufficient releasing of siRNA after uptake.

For efficient silencing effect of target mRNA, sufficient siRNA needs to be delivered to the cytoplasm of the target cells; this is different from plasmid which needs to be delivered to the nucleus. The nanoparticles should meet the requirement when exposing to the complicated *in vivo* environment, a series of delivery obstacles should also need to be conquered to reach the target site (as shown in Figure 2): firstly, the nano vector should be stable enough before use and have the ability to shield from nonspecific uptake of reticuloendothelial system. These nanoparticles should have long half-life time and could recognize the disease cells with high specificity when approaching the target site, which may enable them to be taken in by these cells (Nguyen et al., 2008; Whitehead et al., 2009). Secondly, these nanoparticles should be easy to escape from the endosome (or by other biological pathways) and to release enough siRNA into the cytoplasm. Also, the released siRNAs should be combined with Dicer enzyme to form RISC and cleave the target mRNA to induce the desired silencing efficiency. These nanoparticles should have low toxicity and immunogenicity to the human body for further clinical applications (Fattal and Bochot, 2006; Yang et al., 2013). Till now, the difficulty of siRNA delivery is that these oligonucleotides need to be rationally and precisely designed to conquer quite a lot of challenges along their delivery pathway as described in the above research work for their application. PEGylation could offer a mechanism to shield from nonspecific uptake, conjugating various types of targeting ligands due to the easiness of PEG functionalize as well as provide sterical stability of lipid-based nanoparticles. Thus, how to utilize these advantages of PEG is very critical for rational design of the lipid-based nanoparticle for desired behavior after administrated.

## Different Types of PEGylated Lipid-Based Nanoparticles

**FIGURE 3:**
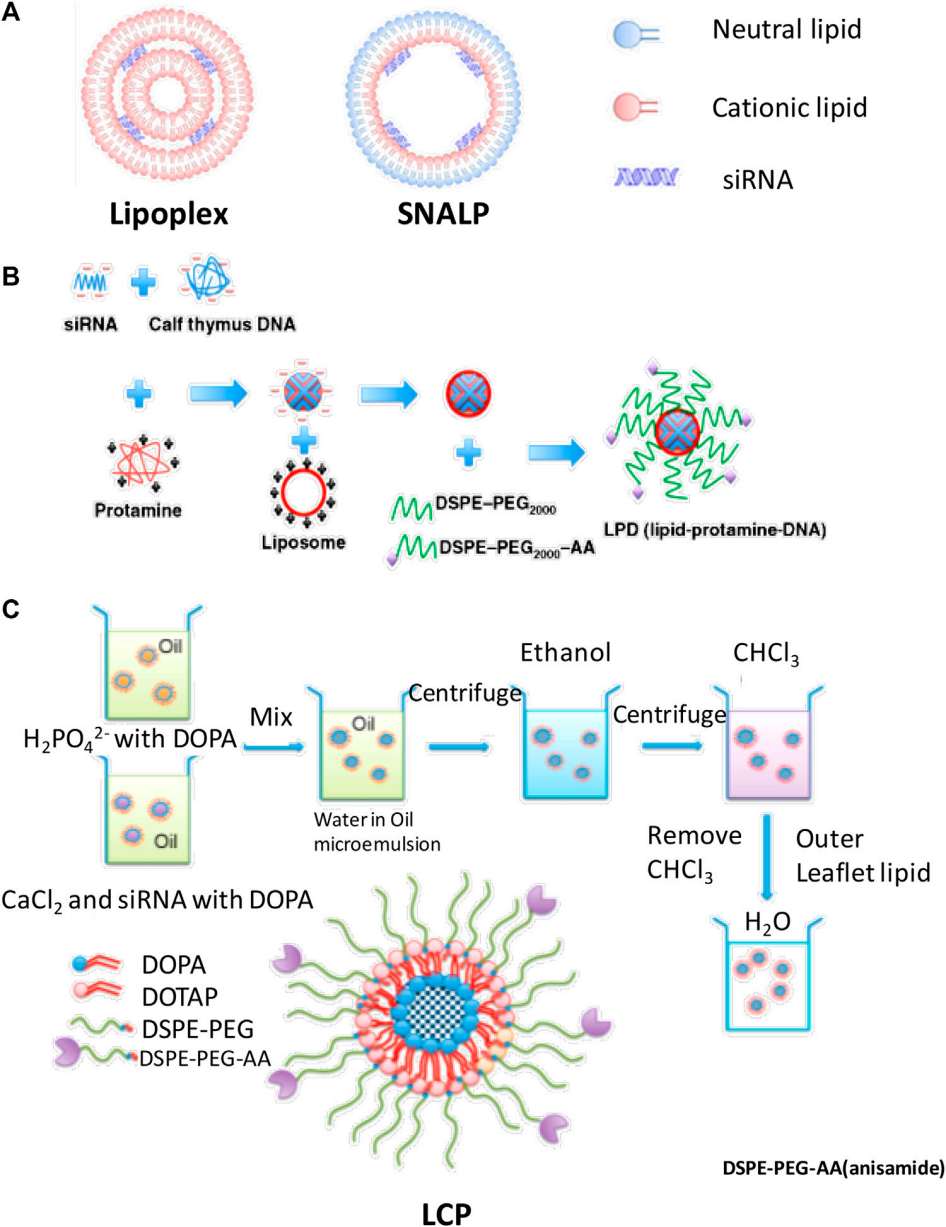
The structure of **(A)** lipoplex and SNALP, **(B)** LPD, and **(C)** LCP, and formulation process of **(B)** lipopolyplex and **(C)** LCPⅡ (Xia et al., 2015) (Kumar et al., 2015).

To achieve efficient delivery of siRNA to the cytoplasm, various types of lipid-based nanoparticles were designed in recent decades and some of them had led to clinical trials (Whitehead et al., 2009; Semple et al., 2010; Kotelianski et al., 2016). In this review, several kinds of lipid (most of them were cationic lipid)-based delivery vesicle which also involved in modification of PEG were introduced in this study; some structures of lipid-based nanoparticle are as shown in Figure 3.

### Lipoplex

The PEG modified liposome and nucleic acid complex can be formed by electrostatic interaction of anionic nucleic acids with cationic liposomes by simply mixing cationic lipid bilayers and siRNA at expected ratios as shown in Figure 3A (Zhang et al., 2005; Huang, 2009). Lipoplex is easy to be formulated and can transfect genes in cultured cells. However, the PEG on the surface of nanoparticle may hinder the endosome escaping *in vivo* and the fraction of PEG coupled on the surface of the lipid-based nanoparticles was limited to maintain their physical stability (Huang, 2009). Also, the extra positive charges of the surface of the lipoplex could be shielded by PEG, rather than neutralized, is still a concern for systematic delivery of these nucleic acids (Sakurai et al., 2007; Muñoz-Úbeda et al., 2012; Kumar et al., 2015). Most of these commercial products such as lipofectamine were only applied *in vitro* in gene delivery studies (Lee et al., 2012).

### Lipopolyplex

Liposome-polycation-DNA (LPD) formed nanoparticles, which contain a highly condensed DNA core of polycation and then encapsulated DNA/polycation cores with lipids around its surface. This formulation process is shown in Figure 3B; siRNA and cationic polymer/or peptide were mixed and condensed to nanoparticles. Cationic lipids were then added to form shell of the LPD nanoparticles. The PEG was incorporated into the liposome to increase its half-life time. The particle size is dramatically decreased compared with lipoplex; however, the physical stability is increased. In some research works, microfluid was applied to formulate lipopolyplex by precisely controlling the flow conditions and mixing process of the reagents at a micrometer scale. Compared with the conventional bulk mixing method, this method can formulate lipopolyplex with more uniformly sized and structured nanoparticles, which may enhance the efficiency for targeting delivery of nucleic acids to cancer cells (Koh et al., 2008; Bathula and Huang, 2010; Koh et al., 2010; Wang and Huang, 2013; Ewe and Aigner, 2014; Zhang et al., 2015; Ewe et al., 2017).

### Lipid–Calcium–Phosphate and siRNA Complex

Lipid–calcium–phosphate and siRNA complex (LCP, classified as LCPІ and LCPⅡ as shown in Figure 3C) nanoparticles incorporate calcium core to promote endosome escaping and siRNA releasing and also function as supporting structure to tolerate up to 10% fraction of PEG on its surface. The principle of LCP nanoparticles delivery system for siRNA is that this vesicle could respond to endosome pH quickly by rapidly dissolving in acidic pH of the calcium phosphate (CaP) (Li et al., 2010; Li et al., 2012). These nanocarriers have some advantages in siRNA delivery such as low sizes (around 50 nm), good stability, high endosome disrupting and siRNA releasing efficiency, and desired targeting efficiency (Chen et al., 2015; Petrilli et al., 2016; Tang et al., 2018). Both of LCPІ and LCPⅡ can achieve high transfection and silencing efficiency compared with LCPІ (Alexis et al., 2008; Li et al., 2012); the design of LCPⅡ with asymmetric lipid bilayer coating permits variety of lipids to be used as out leaflet could provide feasibility to control the pharmacokinetics performance of these lipid-based nanoparticles for enhancement of siRNA delivery (Alexis et al., 2008; Li et al., 2010; Li et al., 2012).

### PEG Cleavable Lipid-Based Vesicles

Conjugation of PEG with proper density can improve steric stability of the nano carrier and increase the half-life time. However, PEG on the surface of the nanoparticle may reduce the cell uptake of the nanoparticle. It can also reduce the endosome escaping efficiency because the interaction between the cationic lipids and the endosomal lipids was retarded due to the steric hindrance (Tseng et al., 2009). To address this issue, lipid-based vesicles further modified with cleavable PEG could provide feasibility of PEG to be removed in a desired place. These sensitive bonds could respond to the different condition quickly, such as low pH value, disulfide, and enzyme degradation (Hatakeyama et al., 2007; Tseng et al., 2009; Schultz and Anseth, 2013). One example is the design of lipopolyplexes with matrix metalloproteinase (MMP)-cleavable PEG (PPD/PEG_5k_-MEND), which could show higher tumor accumulation and gene silencing efficiency. With the development of this field, the strategy of PEG cleavable could be widely used in the designing of gene delivery system to improve the ability of targeting and endosomal releasing of siRNA (Tang et al., 2016).

### Stable Nucleic Acid Lipid Particles

The stable nucleic acid lipid particles (SNALPs) were first developed in 2001 (Semple et al., 2001). The structure was comprised of neural lipid as out-layer and cationic lipid as inner layer to form liposomes. The PEG was conjugated to the surface of the SNALPs to improve the half-life time. Due to the electrostatic interaction between cationic lipids and nucleic acids, siRNA are loaded in the interior of liposome (Wilner and Levy, 2016). This electrostatic interaction could provide the mechanism to achieve high encapsulation rate. This technology is one of the most used lipid-based siRNA delivery methods, which could reduce the immunosimulatory effect with desired gene delivery efficiency after multiple injections (Huang and Liu, 2011).

### Lipid Nanoparticles

Lipid nanoparticles (LNPs) which contain siRNA and ionizable cationic lipids have proven to be effective in delivering gene materials to target sites. Some of them were proven to be effective in clinical trials (Oliveira et al., 2019; Xin et al., 2019). The ionizable cationic lipids such as heptatraconta-6,9,28,31-tetraene-19-yl 4-(dimethylamino) butanoate (Dlin-MC3-DMA) could provide a mechanism (acid dissociation constants below seven) to positively charge at low pH to entrap siRNA and keep neutral surface charges during circulation. Research works on the formation and morphology of LNPs during formulation process were investigated. It proposed that siRNA is first sandwiched between closely apposed lipid monolayer at low pH and then trapped when pH value rose (Kulkarni et al., 2018). Helper lipid such as distearoylphosphatidylcholine (DSPC) formulated in lipid nanoparticles has proven to be vital to the stable encapsulation of siRNA (Kulkarni et al., 2019; Ramezanpour et al., 2019).

### Others

Lipid-based nanoparticles have the potentials to be further used in clinical treatment. siRNA delivery and variety of lipid-based nanoparticles are developed in recent years. It is reported that mono-disperse precise carriers assemble by using the forming materials which conjugated two oleic acids to cationizable oligominnoamide, and the targeting anchor atherosclerotic plaque-specific peptides can couple to the end of the synthesized T-sharp configuration lipo-oligomers to recognize the targeting cell. Several apoptotic peptides are selected to conjugate to the siRNA by disulfide linkage to enhance apoptosis of the diseased cells (Jie et al., 2019). Lipid-based nanoparticles with low burst release and high encapsulation rate were developed by incorporation of lipophilized TNF-α siRNA into solid lipid-based nanoparticles. The TNF-α siRNA was mixed with biocompatible cationic lipid 1,2-dioleoyl-3-trimethylammonium-propane (DOTAP), the nanoparticle could achieve 90% encapsulation rate and reduce the burst release below 5%. Mouse model with collagen-induced arthritis was used to investigate the efficiency of this siRNA loaded nanoparticle. The results showed that after the treatment, paw thickness, bone loss, and histopathological scores were significantly reduced. According to the animal experiment, the lipid-based nanoparticle could deliver siRNA to the chronic inflammation sites and provide new method for treatment of arthritis (Aldayel et al., 2018). Some other modified lipids as well as PEG were also developed for the delivery of siRNA, such as lipopolymer, derived from lipid α-linolenic acid (αLA)-modified low molecular weight polyethylenimine (PEI), which was synthesized and used to deliver BRC-ABL siRNA (Valencia-Serna et al., 2018). The pH sensitivity, enzyme cleavable, or other nano vesicles were also developed to respond to the different biological situations for efficient delivering and releasing of siRNA.

## Different Types of Lipids Used for Formulation of siRNA

### Cationic Lipid

The lipids used for formulation of siRNA include cationic lipid, neutral lipid, and anionic lipid (Wang and Huang, 2013b). Cationic lipid was commonly used to encapsulate siRNA due to the electrostatic interaction. Based on the head groups charges, cationic lipid can be classified into monovalent aliphatic lipids [e.g., N-(1-(2,3-dioleyloxy) propyl)-N,N,N-trimethylammonium chloride (DOTMA)], multivalent aliphatic lipids with head groups containing several amine functions [e.g., dioctadecylamidoglycylspermine (DOGS)] and cationic lipid derivatives [e.g., 3β-(N-(N′,N′-dimethylaminoethane)-carbamoyl) cholesterol (DC-Chol)]. Cationic lipid could also help nanoparticles to interact with negatively charged cell membrane to facilitate endocytosis. However, the positive charges could also induce nonspecific absorption (Wang and Huang, 2013b). PEGylation of the lipid can shield the positive charges with increased steric stability. Ionizable aminolipid such as DLinDMA was pH sensitive (neutral surface charge at physiological pH and protonated in endosome) and highly effective for *in vivo* transfection (Semple et al., 2010).

### Anionic Lipid

Anionic lipid-based delivery systems were also investigated to avoid unfavorable positive charges. They are usually formulated together with protamine or synthetic cationic polymer such as PEI. These polycations may condense negatively charged siRNA to nano-sized particles. Then, the lipids such as 1,2-dioleoyl-sn-glycero-3-phosphate (DOPA), 1,2-dioleoyl-sn-glycero-3-phosphoethanolamine (DOPE), and cholesterol forming anionic liposomes could coat these positively charged siRNA/polycation complex core to assembly lipid membrane on the surface. This method could reduce the surface charges to avoid toxicity (Chen et al., 2010).

### Neutral Lipids

Neutral lipids such as dioleoylphosphatidylcholine (DOPC) liposome need series of processing with limited loading efficiency (Landen et al., 2005). The development of encapsulation of siRNA with neutral cytidinyl lipid/cationic lipid and PEGylation was a promising method for delivery of siRNA. Nucleolipids which consisted of a nucleoside/nucleotide as hydrophilic head and two fatty hydropholic tails present as a promising strategy to be applied in siRNA. They can self-assemble or encapsulate oligonucleotides through nucleobase interaction. To avoid the toxicity invoked by using of cationic lipids, the interactions between nucleobases, such as π-stacking, which is a special interaction to lead formation of DNA helix structure and hydrogen bonding, were used to form nucleic acid lipid nanoparticles (Zhou et al., 2020b). To increase the half-life time and avoid nonspecific interaction, PEG was incorporated in this system. Gemini-like cationic lipid dioleoyl-3,3′-disulfanediylbis-[2-(2,6-diaminohexanamido)] propanoate (CLD) and neutral cytosine-l-yl lipid 2-(4-amino-2-oxopyrimidin-1-yl)-N-(2,3-dioleoyl-oxypropyl) acetamide (DNCA), a useful neutral transfection material for nucleic acid which enables the oligonucleotides to bind *via* H-bonding and π-π stacking with relative low toxicity, were applied to encapsulate 3′,3″-bis-peptide-siRNA (pp-siRNA) to form nanoparticle. DSPE-PEG-cyclic Arg-Gly-Asp (cRGD) was post-inserted to this nanoparticle to improve the ability of recognize target cells express integrin α_v_β_3_ to accumulate in tumor tissue with high specificity. The result shows that this nano carrier could also help siRNA to escape from lysosome to avoid siRNA degradation (Zhang et al., 2019). By the multiple forces such as π-stacking, H-bonding, and also electrostatic force between siRNA and lipids, the neutral cytidinyl lipid DNCA and CLD formed siRNA nanoparticle with further modification of PEG2000-DSPE for delivering siMB3 to specific silencing of BRAF^V600E^ mRNA. The targeting and antitumor efficiency was increased due to the slight negative zeta potentials and uniform sizes of the nanoparticles. The combination strategies of DNCA/CLD lipid could offer a promising method for oligonucleotide delivery for their further clinical therapies (Zhou et al., 2020a).

## Challenges and Strategies

### Involving in Usage of Cationic Lipids in Most of the Lipid-Based Nanoparticles

Cationic lipids were most used in the formulation of lipid-based nanoparticles in the delivery of siRNA. Most of the nanoparticles are comprised of cationic lipids, neutral lipids, and gene materials in oligonucleotides delivery due to the electrostatic interaction of cationic lipids and oligonucleotides can provide feasibility to encapsulate these nucleic acids. Cationic lipids help the lipid-based nanoparticles loaded with siRNA to be absorbed on the surface of target cells based on the positive charges and to escape from endosomes after being trapped (Filion and Phillips, 1997; Aberle et al., 1998; Soenen et al., 2009). However, cationic lipids complexed with siRNA, known as lipoplex, are not stable enough to circulate after administration. Most of the cationic lipid-based nanoparticles are prone to evoke toxicity because of using cationic lipids and easy to aggregate with serum components due to extra positive surface charges (Gebeyehu et al., 1994; Scheule et al., 1997; Lv et al., 2006). Several strategies were adopted such as surface modification of polyethylene glycol (PEG) to shield the positive charges to enhance its safe traveling after administration, but the incorporation of PEG may lead the poor physical stability of nanoparticles, and the proportion of PEG is limited. Some novel lipids were identified and the library approach was built to expand the diversity of cationic lipids to be suitably used in siRNA delivery. One example is synthesis of lipid-like materials which are incorporated with epoxide and oligoamine groups. These lipid-like materials, also known as lipoids, can be mixed with neutral lipids to form nanoparticles to encapsulate siRNA. A series of epoxide-derived lipidoids were synthesized to build a library by the method of high throughput screening. One advantage of this method is that the reactions were typically completed within 3 days without further purification for the following experiment. According to this synthetic strategy, 126 kinds of lipidoids were developed, and the best lipid-like material named C12–200 was identified with desired gene silencing rate at 0.03 mg/kg in nonhuman primates. This method provided an ideal strategy to expand the diversity of lipids for nanoparticle formulation to achieve the high delivery efficiency of siRNA (Kevin et al., 2010; Wang and Huang, 2013a; Uemura et al., 2019; Li et al., 2020; Meel et al., 2020). Tailoring liposome membrane to mimic cell components could also increase delivery efficiency with controllable toxicity. It is reported that significant, dose-dependent increase of efficacy and cell-type specific viability were observed by usage of diacylglycerol (DAG) and phosphatidylserine (PS), which could naturally influence vascular cell function. This method offered a natural strategy for high efficacy delivery of siRNA (Samuel et al., 2018)

### Circulating and Targeting Delivery of Lipid-Based Nanoparticles

After formulating siRNAs with lipid forming nanoparticles, which should be stable enough before application *in vivo*, as mentioned above, most of these lipid-based nanoparticles were administrated systematically, so how to keep these nanoparticles stable to avoid releasing siRNA before reaching the target place and help these nanoparticles to release in the target site are the key factors. The common strategies are the modification of PEG on the surface of nanoparticles and optimization of the sizes and distribution of these nanoparticles to help with long half-life *in vivo*. For disease cell reorganization, the targeting molecules were selected and modified on the surface of lipid-based nanoparticles to enhance the attachment of the targeting cells.

#### PEGylation

**FIGURE 4:**
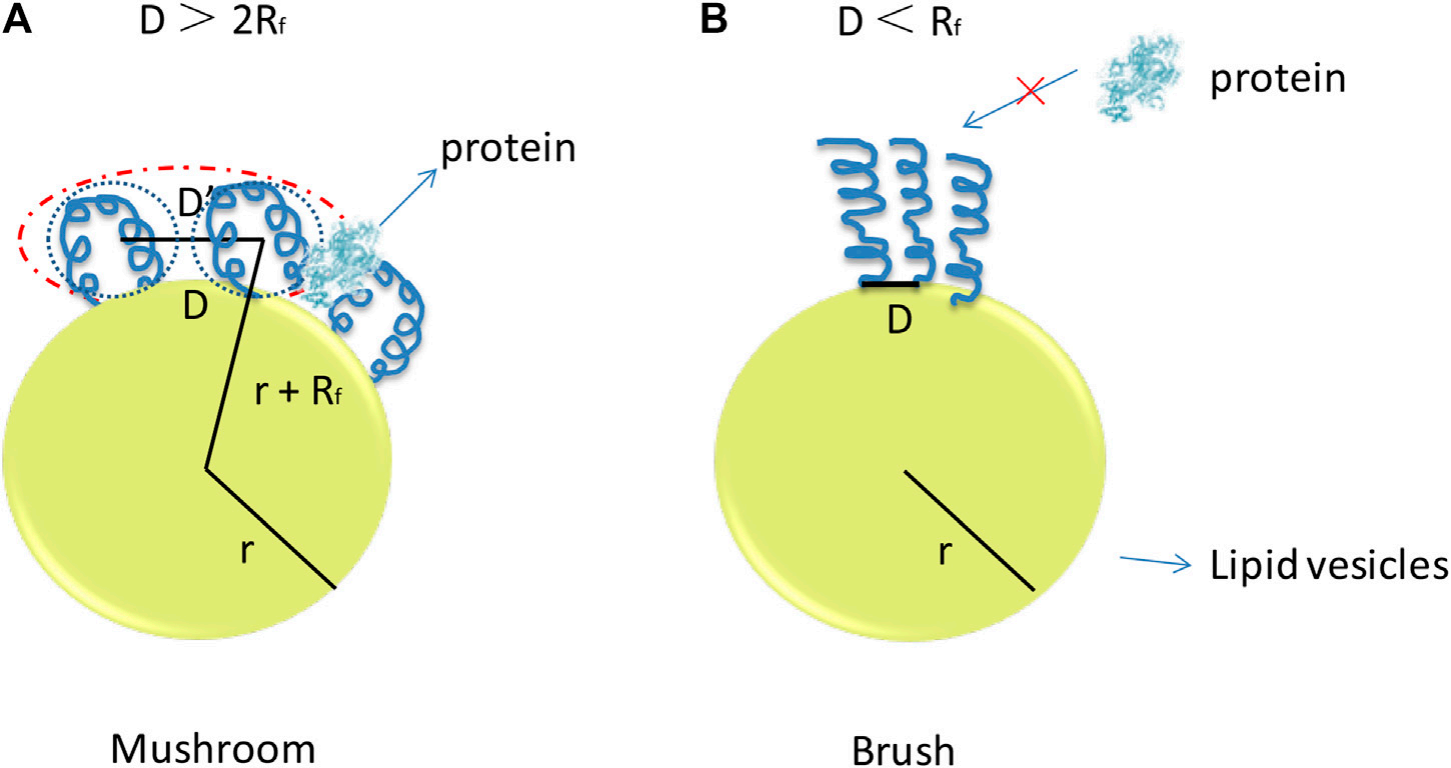
Putative PEG conformation regimes with respect to the polymer concentration in **(A)**, the bilayer, and **(B)** the curvature of the bilayer of the lipid-based nano-carriers. The curvature is high and mushroom of PEG on the surface of the nanoparticles will form when the value of the D > 2*R*_F_. The brush-like structure with relative low curvature of PEG molecule will appear when the value of the D ＜ *R*_F_.

PEG is usually conjugated to lipid molecules with covalent bond and mixes with other lipids to form lipid-based nanoparticles (Tseng et al., 2009; Kumar et al., 2014; Ball et al., 2018). The chain mobility of PEG molecule can provide hydration effect during circulation to avoid nonspecific adsorption of tissues and components in body fluid. This method helps nanoparticles to prolong circulation time and avoid uptake by RES for safe traveling *in vivo*. However, the increased proportion of PEG on the surface of the lipid-based nanoparticles may reduce the mechanical stability and induce the dissociation. PEG can form a mushroom conformation or a brush-like conformation with different degree of polymerization (*N*), monomer size (*a*), and distance of two grafting sites, which can be described as the radius of the random coil (*R*_F_ value, which can be defined by Flory dimensional formula as shown in Eq. 1) and D value (as shown in Figure 4). If D > 2*R*_F_, the curvature is high and mushroom of PEG on the surface of the nanoparticles will form. The brush-like structure with relative low curvature of PEG molecule will appear when the value of the D ＜ *R*_F_ (Kenworthy et al., 1995; Marsh et al., 2003; Moghimi and Szebeni, 2003; Kim et al., 2010).

$$R_{\text{F}\;}=aN^{3/5},$$

where *R*_F_ is the radius of the random coil; *a* is the monomer size; and *N* is the degree of polymerization.

The density of the PEG on the surface of lipid-based nanoparticles is critical for the design of these nano vectors. With increase of the density of grafted PEG on the surface of liposomes, the half-life time was also increased, which attributed to the reduced nonspecific absorption of the protein and RES uptake (Moghimi and Szebeni, 2003). Low incorporation of PEG could not fully protect the nanoparticles to interact with serum proteins because PEG is unable to provide ideal steric stabilization for nanoparticles. However, density of PEG on the surface of lipid-based nanoparticles is limited, high mole fraction of PEG may induce the poor stability of their structure, and lipid bilayer can tolerate about 5–6 mol% of PEGylation commonly. So, the ratio of PEG in lipid bilayer is normally below 5–6% for mechanical stability concern. It is reported that stable liposomes with 4–10 mol% PEG with a molecular weight of 2,000 or larger are typically used in commercial applications. To solve this, liposomes with supporting core such as CaP were incorporated to improve the stability to tolerate more PEG on its surface. It has reported that with the supporting of lipid core forming materials, the fraction of PEG can reach 10% for their mechanical stability consideration (Li and Huang, 2009). This could offer a feasibility to balance the stability and long circulating time after being administrated.

Meanwhile, other issues should also be taken into consideration for PEG modification. The incorporation of PEG on the surface of the nanoparticles can reduce the attachment of the vesicle with the membrane of target cells and also reduce the endosome escaping efficiency. Releasing of siRNA in cytoplasm is thus retarded. To avoid the PEG dilemma, the molecular weight of 2,000 was proven to be good compromise between the enhanced half-life time and efficient delivery strategy (Chen et al., 2019). The de-PEGylation in the target site could be one solution to achieve the efficient silencing of siRNA. The nanoparticles were modified with PEG during the formulation process with proper ratio to maintain the physical stability and hold the ability to reduce the interaction with protein by nonspecific interaction and then de-PEGylation when finding the targeting cells before uptake to enhance the uptake and release of siRNA. To solve this problem, a PEG clearable strategy was adopted to remove the PEG from the surface of the nanoparticles by enzyme degradation, reducing agent (such as disulfide) or incorporation pH sensitive bond to respond to the change of environment (Tseng et al., 2009). Forming of a protein corona in biological fluid after administration could also influence the delivery efficiency of lipid-based nanoparticles for gene medicines. PEG polymer chain length could affect the formation of protein corona, which was proven by quantitatively investigated protein adsorption on nanoparticle with/without PEG modification. The protein human serum albumin (HSA) penetrates and resides within the PEG layer of the nanoparticles with high PEG surface grafts. While nanoparticles without PEG modification, the radius increased ∼3 nm. It consists with formation of a monolayer of HSA on the surface of nanoparticle (Pelaz et al., 2015). It has also been proven that the lipid nanoparticles with similar core composition show different transfect efficiency in HepG2 cells in the presence of fetal bovin serum (FBS) with different lipid carbon chain length (C14-PEG, C18-PEG) and molar ratios. The presence of proteins inhibits uptake of lipid nanoparticles formulated with C18-PEG at ratio of three but facilitates uptake of lipid nanoparticles formulated with C14-PEG (Chen et al., 2019). All of these research works could provide information for rational design of lipid-based nanoparticles.

The increased use of PEGylated therapeutics has proven to result in unexpected immune-mediated side effects. It is reported that anti-PEG antibodies produced by the immune system could specifically recognize and bind to PEG. The appearance of these anti-PEG antibodies has proven to be associated with reduced therapeutic efficacy and increased adverse effects (Garay et al., 2012; Verhoef et al., 2014; Zhang et al., 2016). The PEG modified liposomes have also been reported to stimulate anti-PEG antibody generation. The repeated injection of small liposomes containing 20% PEG-PE liposomes has proven to generate anti-PEG antibodies. The IgG was detected to have an anti-PEG activity (Sroda et al., 2005). It is also found that repeat injection may induce “accelerated blood clearance phenomenon.” The major pre-treated serum protein binding to PEGylated liposomes was IgM and causes subsequent complement activation to accelerate clearance (Ishida et al., 2006).

#### Particle Size Distribution and Surface Characteristics

Particle size distribution and surface characteristics are also factors to affect the nature of lipid-based nanoparticles for their distribution (Abra and Hunt, 1981; Rozenberg et al., 1982). It is reported that particle sizes and distribution have an important influence on their performance *in vivo*. Nano vectors with the size of less than 6 nm are likely to be eliminated after intravenous administration. Particles with diameter around 150–300 nm are easy to distribute in the liver and spleen; other larger molecules are prone to be taken in by monomolecular phagocytic system. In recent work, formulation of lipid particles with narrow size distribution was investigated to obtain the desired pharmacokinetics behavior (Gao and Huang, 2013).

Zeta potential is also an important issue to increase the half-life time. The best advantage of cationic lipids used in formulating siRNA nanoparticles *in vivo* is that the positively charged lipids can form complex with the negatively charged siRNA in high efficiency. The complex can be easily taken up by cells due to the extra positive charges on surface. However, the positive charges may interact with serum proteins and thus induce larger particle size during the systematical circulation. This is one reason to evoke the unwanted immune response or side effect, so an ideal lipid-based nanoparticle should be designed with proper and uniform sizes, low surface charges, and required shapes (Gabizon and Papahadjopoulos, 1992).

#### Surface Targeting Modifications and Other Strategies

To realize the targeting delivery of siRNA to diseased cells, different types of molecules such as chemical molecules, peptides, antibodies, and aptamers were immobilized on the surface of the lipid-based nanoparticle as anchors to help these nano vesicles to find the targeting cells. The modification of the selected targeting molecule can bond with receptors over-expressed on the surface of the disease cells with high affinity and thus induce the attachment of the siRNA nanoparticles (Song et al., 2005; Mcnamara et al., 2006; Peer et al., 2008; Wheeler et al., 2011; Kwok, 2013; Khatri et al., 2014). The targeting efficiency varies with optimized type, density, and orientation of the targeting molecule (Ge et al., 2015). For example, folate was usually used as target molecule and modified on lipid-based nanoparticles for tumor killing. It is easy to be conjugated to lipids or PEG-lipid and mixed with other lipids to form nanoparticles. It is reported that complexing siRNA with novel azido-functionalized sequence-defined cationizable lipo-oligomer, which containing two cholanic acids attached to an oligoaminoamide backbone, could be further modified with folate as targeting molecule. By using double click for the modification, these nanoparticles can achieve desired bio-distribution and intracellular delivery of siRNA (Klein et al., 2018). Aptamers or antibody fragments were also incorporated to nano vesicles due to their high binding efficiency and specificity. Aptamers are a type of synthetic oligonucleotides with three dimensional folding structures. They have emerged as targeting ligands and widely used in drug delivery system which can display a high affinity in recognizing of certain specific receptors with low toxicity and immunogenicity (Liu et al., 2014; Shiao et al., 2014; Xu et al., 2018). Anti-CD44 aptamer has been incorporated into liposomes as targeting ligands to encapsulate protamine condensed siRNA and has shown efficient gene silence efficiently to give evidence in the possibility conjugation of aptamer as moieties to target delivery of gene materials (Alshaer et al., 2017). Some proteins such as transferrin could be also used as target molecule to be modified on the surface to siRNA loaded liposomes. It could recognize tumor cells to facilitate cell attachment of lipid-based nanoparticle with high efficiency (Mendon et al., 2010). Peptides, such as RVG, a 29-amino-acid peptide which was derived from rabies virus glycoproteins, could be immobilized on the surface of siRNA loaded liposome and function as the target ligand to the brain. It has proven that the modified liposome holds the ability to across the blood brain barrier with high penetration efficiency. It could provide a promising method to develop new gene delivery systems (Tao et al., 2012; Meng et al., 2018). Other strategies were also used to improve the targeting efficiency. For example, pH sensitive lipids were adopted to mix with other components to respond to the pH changes of different biological environment and thus enhanced the targeting efficiency of the nucleic acids. A dioleylphosphate-diethylenetriamine conjugate (DOP-DETA) which consists of a pH-responsive triamine and unsaturated fatty acid was designed and accelerated membrane fusion. This kind of liposome was proved to deliver siRNA into the cytoplasm with high efficiency and induce silencing efficiency at a low siRNA concentration (Sako et al., 2019).

### Endosome Escaping Efficiency

**FIGURE 5:**
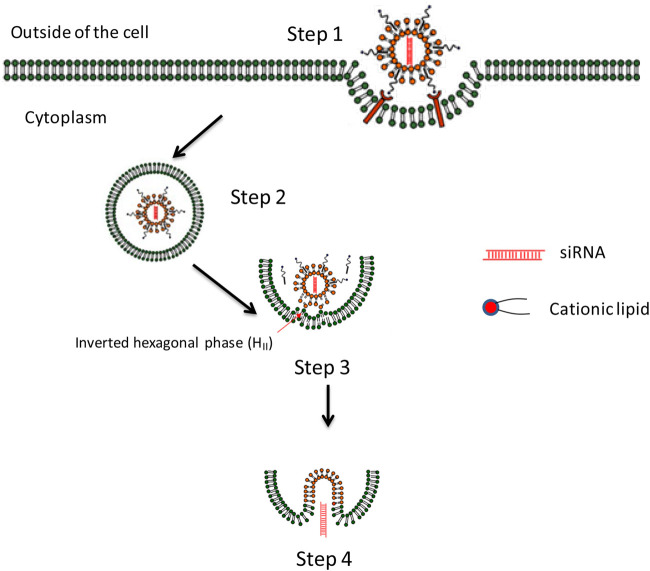
The endosome escaping mechanism of lipid-based nanoparticles lipoplex for delivery of siRNA. *Step 1*. Cell attachment and uptake. *Step 2.* Trapped in endosome. *Step 3.* Formation of ion-pair between cationic lipid from lipoplex and membrane lipid. Endosome membrane destabilizes due to the formation of the inverted hexagonal phase (HII) which may provide possibility for siRNA releasing. *Step 4.* Releasing of nucleic acid to cytoplasm and inducing related gene silencing effect (Tang et al., 2018).

After attachment, the nanoparticle is taken in by cells and releases from both endosomes and nano vesicles with sufficient quantities to form RISC and induce related silencing effect. Unlike cationic polymer formed nanoparticles, which are supposed to rely on the “proton sponge” effect to enhance endosomal release, lipid-based vesicles depend on the nature of lipid bilayer to help with endosome escaping. The endosome escape mechanism and process for the lipid-based siRNA nanoparticles are shown in Figure 5. Most of the lipid-based siRNA nanoparticles are involved in the use of cationic lipids; these cationic lipids may form ion pairs with the ionic lipids from the endosome membrane. The stability of endosome membrane is thus interrupted and the inverted hexagonal phase (HII) is formed by the cationic lipids from nano vesicles and ionic lipids from endosome membrane. During this process, the endosome membrane is destabilized and the structure of the nanoparticle is dissociated for siRNA releasing. The use of fusogenic lipids such as dioleoylphosphatidylethanolamine (DOPE) can help these lipid-based nanoparticles to facilitate endosome escaping according to the interaction between lipid membrane of both lipid-based nanoparticles and endosomes. The increase of endosome escaping mostly depended on the effort of cationic lipid hydrophobic domain and the fusogenicity of the delivery system (Farhood et al., 1995; Hassani et al., 2005; Heyes et al., 2005). As mentioned before, the conjugation of PEG improves the steric stability and half-life time of nanoparticles during systematic circulation. But it reduces the interaction between the nanoparticle forming lipid and endosome. pH sensitive PEG-lipids were synthesized to formulate with other lipids. The formed lipid-based nanoparticles could maintain stable during the circulation at neutral pH and de-PEGylated below pH 5.5 to promote endosome escaping, for example, the lipid-based nanoparticles formulated by nonlamellar highly fusogenic phosphatidylethanolamine (PE) lipids and pH sensitive PEG-lipids which are conjugated by vinyl ether lipids. When exposing to low pH value, the vinyl ether bond is hydrolyzed to remove PEG block, promoting the dissociation of lipid-based nanoparticles and facilitating the interaction with endosome membrane to induce endosome escaping and siRNA releasing. This solution gives modality for PEG molecule to be “PEGylated” or “de-PEGylated” in different biological situations for advanced delivery of siRNA (Shin et al., 2003).

Cell penetrating peptide or pore forming peptide, such as human immunodeficiency virus (HIV)-derived peptide, can also be used in the design of lipid-based nano-vesicles to help with endosome escaping processing (Kwon et al., 2008; Endoh and Ohtsukhi, 2009; Tseng et al., 2009; Dominska and Dykxhoorn, 2010; Nieva et al., 2012). Most of these peptides can change their conformation responding to the acidity of endosomes and expose their hydrophobic components to strongly face with the endosome membrane. Some other strategies were also adopted to enhance the endosome escape of lipid-based nano vesicles, such as incorporation of cationic core (e.g., protamine), degradable cationic polymer, and also the CaP to enhance the dissociation of nanoparticle facilitating endosome escape by combination of “proton sponge” effect. These methods release sufficient siRNA into the cytoplasm of the target cells and induce silencing effect.

For endosome escaping, the degradation should be taken into consideration. It not only affects the releasing of siRNA but also is related to biocompatibility. It is reported that designing of precise enzymatic cleavable smart lipid-based nanoparticles may provide new solution for enhanced target delivery of siRNA with relative low toxicity induced by materials. To reduce the toxicity of amphiphilic molecules accumulated to lysosomes and other intracellular organelles, the carriers forming materials can be synthesized as cationic lipo-oligomers containing amino acids with precise enzymatic cleavage sites which can be degraded to low toxicity fragments by endolysosomal enzymes to facilitate the excretion. This research work provided possibility for incorporating new motifs in rational design lipo-oligomers to achieve safe and efficient siRNA delivery (Reinhard et al., 2018).

### Potential Toxicity Induced by Nanoparticles

The toxicity of the lipid-based nanoparticles for delivery of siRNA is critical to turn research work to clinical applications. The toxicity of the lipid-based nanoparticles for siRNA delivery is mainly caused by usage of cationic lipids and also the immune response from siRNA (Tan, 2001; Bridge et al., 2003; Sledz et al., 2003; Soenen et al., 2009; Oliveira et al., 2019). siRNA can induce immune response to trigger interferon and inflammatory effect by the toll-like receptors (TLRs) such as TLR3 and TLR7, and the use of lipid-based nano vector may accelerate this activation to evoke unwanted side effects (Dass, 2002; Hornung et al., 2005; Kleinman et al., 2008). Cationic lipids may induce toxicity, and the use of these lipids can increase the zeta potential of formed lipid-based nanoparticles, which results in the nonspecific adsorption of components in serum and uptake by RES. Some cationic lipids can also simulate immune response and affect cell signaling pathways (Vangasseri et al., 2006; Lonez et al., 2008). The structural design of these lipid materials has the potential to improve the safety application of lipid-based nanoparticles for siRNA delivery, and the doses of both siRNA and lipids should be optimized when applied (Perrie et al., 2001; Niculescu-Duvaz et al., 2003; Kuboyama et al., 2019; Lin et al., 2019; Sato et al., 2019).

As discussed above, these factors may affect the targeting delivery efficiency of siRNA after administration. The desired requirements for delivery of siRNA by lipid-based nanoparticle are summarized in Table 1. Based on the principle of these requirements, the delivery system can be precisely designed and further optimized to improve its efficacy of siRNA, which can help them to be applied from bench to beside.

**TABLE 1:** Desired requirements for lipid nanoparticles for siRNA delivery.

	Factors	Requirements
1	Sizes	Smaller size of gene carriers can be beneficial in penetrating tissues and in the cellular uptake. Smaller than 100 nm: necessary to access hepatocytes; 100–200 nm: accumulate in the tumor site through the enhanced permeability and retention (EPR) effect (Huang and Liu, 2011; Xia et al., 2015)
2	Zeta potential	Reduced nonspecific interactions; good particle stability; enhanced cellular uptake and retention; and sensitivity to environmental factors or triggers like pH and temperature (Gilbile et al., 2019)
3	PEGylation	Short PEG (e.g., PEG1K or smaller) cannot notably reduce the adsorption of proteins and extend the circulation time efficiently. Long PEG (e.g., PEG5K or larger) reduces the cellular uptake and the endosomal escape of liposomes. Commonly used PEG2K: ＜5%, coverage ＜ 100%, mushroom-like; 5–15%, full coverage, mushroom-like and brush-like; ＞15%, full coverage, brush-like (Xia et al., 2015)
4	Target ligand	The type, density, and orientation of the target ligand are key factors for targeting efficiency. Further modification with targeting ligands can increase gene silence efficiency but does not significantly affect the pharmacokinetics or biodistribution profiles of the nanoparticles. High amount of targeting ligands may reduce the effect of PEG on the surface of lipoplexes (Huang and Liu, 2011; Ge et al., 2015; Xia et al., 2015)
5	Endosome escaping	PEG removable: diorthoester, hydrazone linker (low pH), disulfide (reducing agents), and peptide (enzyme). Incorporating pH sensitive cores, such as CaP, cationic polymer. (Huang and Liu, 2011). Cell penetrating peptide or pore forming peptide (Gabizon and Papahadjopoulas, 1992; Song et al., 2005; Tseng et al., 2009; Dominska and Dykxhoorn, 2010; Gao and Huang, 2013)

### Clinical Trials

From the webpage of Food and Drug Administration (FDA), more than 30 RNA-based therapies are in clinical trials. Although the development of siRNA drugs experienced a hard time, the approved lipid-based RNA drug patisiran is very inspiring (Adams and Ole, 2018; Buxbaum, 2018). The Dlin-MC3-DMA, a kind of ionizable cationic lipids, was used to form siRNA loaded lipid nanoparticles. Patisiran was applied for treatment of TTR mediated amyloidosis which could reduce the expression of target protein mutated transthyretin (TTR) to 80% at the dose of 0.3 mg/kg every 3 weeks (Ole et al., 2015). It could show good stability in circulation with more than 95% encapsulated siRNA in lipid particles with controllable infusion-related reactions (Simoneide et al., 2020; Zhang et al., 2020). Besides, some clinical development research works were as shown in Table 2. Taking advantage of the lipid-based delivery system, we can optimize nanoparticles to be more suitable for siRNA delivery. How to overcome obstacles and barriers for efficient target delivery of siRNA remains unsolved (Whitehead et al., 2011; Kanasty et al., 2013; Khvorova et al., 2014; Duarte Joao, 2015).

**TABLE 2:** siRNA clinical trials.

Name	Therapeutics	Delivery system	Sponsor	Phase and status
ONPATTRO (patisiran, ALN-TTR02)	TTR-mediated amyloidosis	LNP (DLin-MC3-DMA)	Alnylam Pharmaceuticals	Approved commercialized
PRO-040201	Hypercholesterolemia	LNP	Arbutus Biopharma Corporation	Phase I/II terminated
DCR-PH1	Primary hyperoxaluria type 1	LNP	Dicerna Pharmaceuticals	Phase I terminated
ARB-001467	Hepatitis B	LNP	Arbutus Biopharma Corporation	Phase II completed
TKM-100802	Ebola	LNP	Arbutus Biopharma Corporation	Phase I terminated
TKM-080301	Hepatocellular carcinoma, hepatoma, liver cancer, liver cell carcinoma, neuroendocrine tumors, and cancers with hepatic metastases	LNP	Arbutus Biopharma Corporation	Phase I/II completed
Atu027	Carcinoma and pancreatic ductal	Cationic lipoplex	Silence therapeutics	Phase I/II completed
DCR-MYC	Hepatocellular carcinoma	LNP	Dicerna Pharmaceuticals, Inc.	Phase I/II terminated and has results
ALN-VSP02	Solid tumors	LNP (Dlin-DMA)	Alnylam Pharmaceuticals	Phase I completed and program hold
siRNA-EphA2-DOPC	Advanced cancers	Liposome	M.D. Anderson Cancer Center	Phase I recruiting
ND-L02-s0201	Idiopathic pulmonary fibrosis	LNP and vitamin A	Bristol-Myers Squibb	Phase II recruiting

## Conclusion

Lipid-based nanoparticles are widely used in targeting delivery of nucleic acids and function as a promising platform in the generation of more siRNA drugs. In the last decade, more efforts have been put to the discovery of the mechanisms, delivery pathways, biological barriers and solutions, and methods for evaluating the behavior and performance of the lipid-based nanoparticles. Although great progress has built in lipid-based siRNA delivery system, we still need to put much more effort in turning these research work to practical applications. It is important to learn more about the relationship between “structure—activity” of the lipids to balance the silencing efficiency and biocompatibility and to know more information for potential toxicity and immunity induced by these materials. To realize the clinical applications of siRNA, more efforts should focus on the design of these lipid-based nanoparticles with safe delivery after administration and the ability to get access to various disease cells with high efficiency and specificity. For further applications, we still need to think about how to develop lipid-based siRNA nanoparticles easily. All of these works will be helpful for advancing the siRNA lipid-based nanoparticles to be more suitable.

## Author Contributions

WY conducted the review and XG wrote the draft while BZ and LC revised the manuscript and helped with the figures. All authors contributed to substantial enhancement of the manuscript.

## Funding

This work was financially supported by the National Science Foundation of China (Grant Nos. 81601596, 31770921, and 31971187).

## Conflict of Interest

The authors declare that the research was conducted in the absence of any commercial or financial relationships that could be construed as a potential conflict of interest.
